# Annular Fiber Probe for Interstitial Illumination in Photoacoustic Guidance of Radiofrequency Ablation

**DOI:** 10.3390/s21134458

**Published:** 2021-06-29

**Authors:** Hindrik Kruit, Kalloor Joseph Francis, Elina Rascevska, Srirang Manohar

**Affiliations:** 1 Multi-Modality Medical Imaging, Technical Medical Center, Faculty of Science and Technology, University of Twente, 7522 NB Enschede, The Netherlands; s.manohar@utwente.nl; 2 Biomedical Photonic Imaging, Technical Medical Center, Faculty of Science and Technology, University of Twente, 7522 NB Enschede, The Netherlands; f.kalloorjoseph@utwente.nl (K.J.F.); erascevs@uwo.ca (E.R.)

**Keywords:** photoacoustics, ultrasound imaging, multimodal imaging, interventional imaging, interstitial illumination, radiofrequency ablation, liver treatment, minimally invasive procedures, surgical tool tracking

## Abstract

Unresectable liver tumors are commonly treated with percutaneous radiofrequency ablation (RFA). However, this technique is associated with high recurrence rates due to incomplete tumor ablation. Accurate image guidance of the RFA procedure contributes to successful ablation, but currently used imaging modalities have shortcomings in device guidance and treatment monitoring. We explore the potential of using photoacoustic (PA) imaging combined with conventional ultrasound (US) imaging for real-time RFA guidance. To overcome the low penetration depth of light in tissue, we have developed an annular fiber probe (AFP), which can be inserted into tissue enabling interstitial illumination of tissue. The AFP is a cannula with 72 optical fibers that allows an RFA device to slide through its lumen, thereby enabling PA imaging for RFA device guidance and ablation monitoring. We show that the PA signal from interstitial illumination is not affected by absorber-to-surface depth compared to extracorporeal illumination. We also demonstrate successful imaging of the RFA electrodes, a blood vessel mimic, a tumor-mimicking phantom, and ablated liver tissue boundaries in ex vivo chicken and bovine liver samples. PA-assisted needle guidance revealed clear needle tip visualization, a notable improvement to current US needle guidance. Our probe shows potential for RFA device guidance and ablation detection, which potentially aids in real-time monitoring.

## 1. Introduction

Hepatocellular carcinoma (HCC) and colorectal liver metastases (CLM) are tumors with high occurrence in the liver [1,2]. Surgical resection is the mainstay of treatment with curative intent [2,3]. However, the tumor is considered to be unresectable in the presence of multiple tumors, vasculature invasion, too extensive liver disease, and co-morbidity. In patients with CLM, only 10–20% of them qualify for resection [1,4]. For these unresectable cases, minimally invasive percutaneous radiofrequency ablation (RFA) is an effective outcome for tumors < 3 cm [1,3,4,5]. This technique has several benefits compared to resection: reduced morbidity, shorter hospitalization times, lower costs, and less damage to surrounding tissue [6].

During RFA, multiple electrodes, called tines, are deployed from the applicator inside the tumor. A high-frequency alternating current is then applied, creating frictional heating in tissue close to the tines. This heat diffuses through the tissue, creating an ablation zone in which high temperatures between 50 and 100 °C cause coagulation necrosis, destroying the tumor cells [7]. Despite its success, RFA has a higher probability of tumor recurrence compared to liver resection due to incomplete ablations [8,9]. Percutaneous RFA is subject to varying recurrence and local tumor progression rates, being 21–43% [10,11,12]. Factors contributing to successful treatment are (1) locating the tumor and blood vessels, (2) accurate placement of the RFA device, and (3) delineating the extent of the ablation zone [8]. Thus, accurate image guidance is essential for proper device targeting and assessment of ablation success. Incomplete ablation frequently occurs with tumors near large blood vessels since they dissipate heat from the ablation region (heat sink effect) [13]. Locating the tumor and its proximity to blood vessels is an important step in achieving complete ablation [8].

Both computed tomography (CT) and ultrasound (US) imaging are widely used for image guidance during RFA, having comparable outcomes regarding safety and efficacy [14]. Despite the fact that CT delineates the ablated region more accurately, US is more commonly used as it is more convenient, low cost, highly available, and a real-time imaging modality [14,15]. However, US imaging is subject to shortcomings. Formation of gas bubbles occurs during ablation, resulting in hyperechoic areas in the image that do not necessarily correspond to the ablated zone, leading to undertreatment of some areas [16,17]. In addition, needle visualization in tissue with US is not straightforward due to artifacts and out-of-plane reflections, making it challenging to accurately guide needles to the target [18,19].

A recently proposed strategy is using photoacoustic imaging for guiding the RFA device (needle) toward the tumor and monitoring ablation [20,21]. In photoacoustics (PA), tissue is illuminated by short nanosecond laser pulses that are absorbed by tissue constituents; the absorption creates a temperature rise, causing thermoelastic expansion. The expansion generates a pressure wave (PA wave) with MHz frequencies, such that a conventional US transducer can detect them [22,23]. The amount of PA pressure is proportional to the wavelength-dependent absorption of light, enabling optical distinction between tissue chromophores when tissue is illuminated at specific wavelengths. The RFA needle, native (untreated) and ablated liver [20], blood [24], and liver tumors [25,26] differ in optical properties, enabling detection and distinction with PA imaging. Therefore, this technique shows the potential to guide the needle toward the tumor, depict the location of blood vessels and monitor ablation during RFA of the liver.

Guiding needles with the aid of PA imaging for clinical intervention has shown to be superior to US imaging alone [20,27,28,29]. Several works have focused on the PA detection of ablated tissues in the liver [20], kidney [30], and heart [31,32,33]. A limitation in most of these works is the shallow PA imaging depth due to the low penetration depth of light. Typical tissues have an imaging depth of approximately 4 cm [34], while a dark organ, such as the liver, the optical penetration depth is only 3–7 mm [35]. A way to overcome this limitation is by bringing light percutaneously into tissue near the region of interest, as was first introduced by Piras et al. [28] and termed interstitial illumination for PA imaging by Bell et al. [36]. This approach has since been applied in several applications [34].

We previously showed, for the first time, PA imaging guidance of an RFA device in ex vivo tissues such as the liver and ablation visualization, using extracorporeal illumination [20]. To overcome the low penetration depth of light, we developed a probe for interstitial illumination. Preliminary results with this probe showed that PA imaging was superior to US imaging in visualizing the RFA needle and an optically absorbing target [37]. By incorporating optical fibers on the surgical tool tip, light together with the surgical tool can be directed to the region of interest, as first shown by Eddins and Bell (2017) [38]. Our probe has a similar approach, having multiple optical fibers in an annular arrangement on a hollow needle whose lumen accommodates an RFA device. This integrated approach allows insertion of the RFA device into tissue, while interstitial illumination can be performed for PA imaging. The device, which we henceforth refer to as the annular fiber probe (AFP), was designed to withstand RFA ablation temperatures and illuminates in the visible to near-infrared wavelength region. In this article, we characterize and validate the imaging performance with the AFP. First, a Monte Carlo simulation was conducted to investigate the fluence distribution in tissue and the number of fibers used in the probe. Second, the PA field of view was characterized in a phantom. Third, extracorporeal and interstitial illumination were compared in PA imaging of a target at different depths. Fourth, the device was tested on phantoms and ex vivo tissues to measure the PA imaging capability. These were performed on the detectability of the probe’s tip and RFA needle with its tines in tissue. Next, human donor blood was used to detect a human blood vessel phantom and a tumor-mimicking phantom in the liver. Finally, the ability to visualize an ablation boundary was evaluated.

## 2. Methods

### 2.1. Probe Design and Experimental Setup

#### 2.1.1. Annular Fiber Probe Design

**Figure 1:**
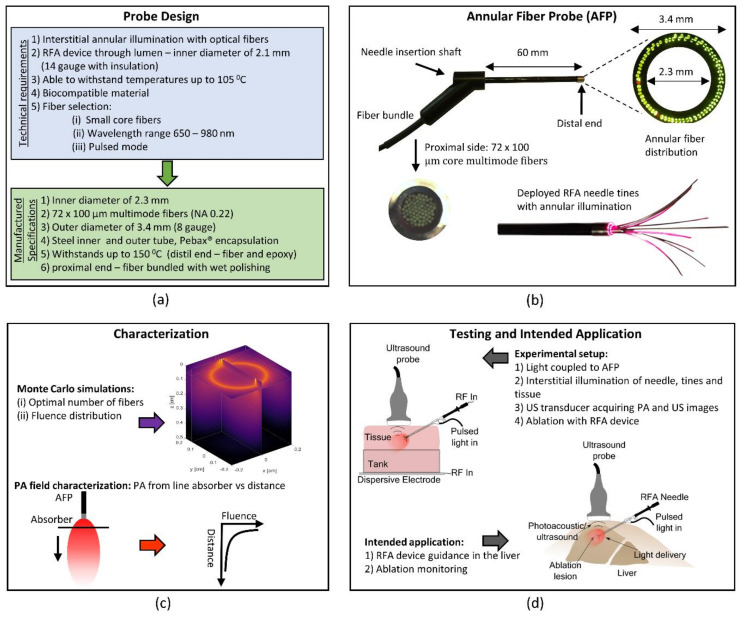
Annular fiber probe (AFP): design, experiments, and intended application. (**a**) AFP design requirements and manufactured specifications. (**b**) Photographs. (**c**) Upper part, Monte Carlo simulations to estimate the fluence distribution of the AFP. Lower part, assessing the photoacoustic (PA) field of view in a liquid optical phantom. (**d**) Upper part, the general setup for testing in ex vivo tissue, allowing combined PA and ultrasound (US) imaging. Lower part, the intended application of the AFP, guiding the RFA needle in the liver and monitoring of the ablation process.

Figure 1 gives an overview of the design requirements and final probe design together with the evaluation and intended application of the AFP. Illumination is achieved by the probe, and the generated PA signals are detected using a conventional US transducer. The probe should be able to hold a typical RFA applicator in its lumen, being at least 2.1 mm in diameter. Typical ablation temperatures are around 105 °C, which the probe should be able to withstand. Optical fibers should be incorporated that allows for pulsed laser light illumination for wavelengths between 680 and 980 nm. To interstitially illuminate tissue while also carrying the RFA device, the fibers should be arranged in an annular fashion at the distal end. The design of the probe was realized at the University of Twente and Art Photonics (GmbH, Berlin, Germany) was responsible for manufacturing it, see Figure 1a. The manufactured probe has a catheter shaft consisting of an outer steel ferrule with a diameter of 3.4 mm, and an inner steel ferrule with a diameter of 2.3 mm. The latter allows sliding through an RFA device. Between the two ferrules, the optical fibers are arranged annularly and glued at the distal end with epoxy (EPO-TEK^®^ 353ND, Epoxy Technology, Billerica, MA, USA), setting the maximum operating temperature to 150 °C. A total of 72 multimode optical fibers with an NA of 0.22 and a core diameter of 100 μm run from the proximal side, where they were bundled and wet polished, to the distal end. The outer shaft of the catheter was encapsulated with Pebax^®^ (Arkema S.A., Colombes, France). This probe is further referred to as the annular fiber probe (AFP). See Figure 1b for pictures of the manufactured AFP and an RFA device slit through its lumen with deployed tines under annular illumination. Characterization of the field of view is schematically shown in Figure 1c. The general setup for PA and US imaging can be seen in the upper part of Figure 1d. The intended application is visualized in the lower part of Figure 1d.

#### 2.1.2. Experimental Setup

For illumination with the AFP, laser light from an optical parametric oscillator (OPO) (versaScan L-532, Spectra-Physics^®^, Santa Clara, CA, USA) was coupled to the proximal side of the AFP. The OPO was pumped by a 10 Hz Nd:YAG laser operating at 532 nm (Quanta Ray Lab 170, Spectra-Physics^®^, Santa Clara, CA, USA). The desired wavelength was selected from the signal output of the OPO (680–950 nm). For surface illumination comparison, light from the OPO was coupled, using a flip mirror, to a multi-output fiber bundle (CeramOptec GmbH, Bonn, Germany). Six fibers from the fiber bundle were placed in a 3D printed US probe holder, having the fibers next to the transducer for surface illumination [20].

The Alpinion Ecube 12R (Alpinion Medical Systems, Dongan-gu, South Korea) is a commercially available US system and was used in research mode for PA and US acquisition. A custom-made sequence was used to acquire US and PA images for real-time visualization and data storage [20]. When the laser sends a synchronization trigger pulse to the Alpinion, data were acquired for combined PA and US imaging. The L3–12 US transducer with a center frequency of 8.5 and 5–11 MHz bandwidth (−6 dB) was used to detect the US and PA signals.

For all experiments, 20 PA and US frames were collected and averaged to increase the signal-to-noise ratio (*SNR*). In most experiments, there was a dynamic range of 30 dB in the PA images and 60 dB in the US images. In addition, to quantitatively compare the visibility of structures within the images, the *SNR* was determined of the tip and structures. For this calculation, the following definition was used: $SNR=20\log_{10}\left(\frac{S_{R}}{S_{n}}\right)$, in which $S_{R}$ represents the mean of normalized pixel values in the region of interest (ROI) and $S_{n}$ represents the mean normalized pixel value of the background signal. The mean ROI pixel value was determined several times per object to create variation in the calculated *SNR*, allowing the standard deviation of these *SNR*s to be interpreted as a measure of accuracy.

### 2.2. Device Testing and Characterization

#### 2.2.1. Monte Carlo Simulations

**Figure 2:**
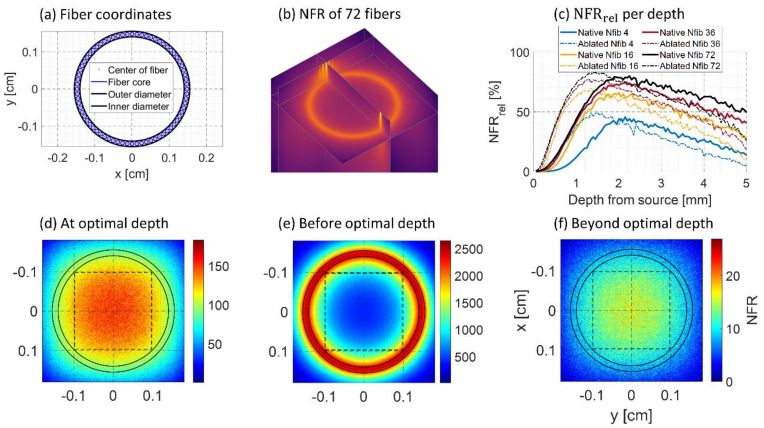
Monte Carlo simulation (MCS) results at 650 nm. (**a**) The location of the fibers used in the MCS. The black circles delineate the inner and outer diameter of the probe. (**b**) 3D normalized fluence rate (*NFR*) of 72 fibers in native liver tissue resulting from the MCS. (**c**) $NFR_{rel}$ per depth for native and ablated tissue simulated for 4, 16, 36, and 72 fibers (Nfib) in the annular ring. (**d**–**f**) 2D slices: at the optimal depth, before optimal depth, and beyond optimal depth. The dashed lines indicate the ROI from which $NFR_{rel}$ was determined.

To understand the fluence distribution as a function of depth from the source and its dependence on the number of fibers mounted in the AFP, Monte Carlo simulations were performed. The open-source MCmatlab toolbox was used for the Monte Carlo simulation (MCS), which is a 3D Monte Carlo radiative transfer equation solver [39]. The software can be fully programmed and executed from MATLAB (The MathWorks, Inc., Natick, MA, USA). The simulation setup comprised of a cube of liver tissue, spanning a space of 4 × 4 × 5 mm (x, y, z) divided into 820 grid points in all directions. From the top surface (XY plane), photons were launched at the fiber locations (see Figure 2a), representing the distal end of the AFP, with 72 fibers being the full occupancy of fiber space. From the simulations, the fluence distribution was determined for several fibers distributed along the annular ring. The fibers had a 100 μm core diameter and launched a Gaussian beam profile of photons with a numerical aperture of 0.22. The fluence distribution was determined for 4, 16, 36, and 72 fibers distributed in the ring. Each fiber launched 3 million photons sequentially. The photons were given wavelengths of 650 nm, 900 nm, and 1050 nm. The simulation used the absorption coefficient $\mu_{a}\;\left[{\mathrm{cm}}^{-1}\right],$ scattering coefficient $\mu_{s}\;\left[{\mathrm{cm}}^{-1}\right]$, anisotropy factor g, refractive index n and wavelength $\lambda \;\left[\mathrm{nm}\right]$ as primary input, and the values chosen were matched with those of native and ablated liver tissue at the corresponding wavelengths (Table 1).

The determined values of $\mu_{a}\left(\lambda \right)$ and $\mu_{s}^{\prime }\left(\lambda \right)$ of native and ablated liver tissue at 70 °C were taken from reference [40], from which $\mu_{s}$ was evaluated as $\mu_{s}=\frac{\mu_{s}\prime }{\left(1-g\right)}$ [42]. Generally, in ablated liver tissue, the absorption is lower, while the scattering is considerably higher compared to the native state. The anisotropy factor g and refractive index n were estimated from references [12,13]. However, no data were found in the literature about the refractive index of ablated liver tissue. Therefore n was considered the same for both tissue types during the simulation. The determined fluence map for a single fiber configuration at one wavelength and tissue state requires 7 GB of data storage. Each simulation took approximately six hours on a computer with an Intel^®^ Core™ i7-7700 3.6 GHz quad-core processor and 47.9 GB of RAM.

#### 2.2.2. PA Field Characterization with an Absorber

**Figure 3:**
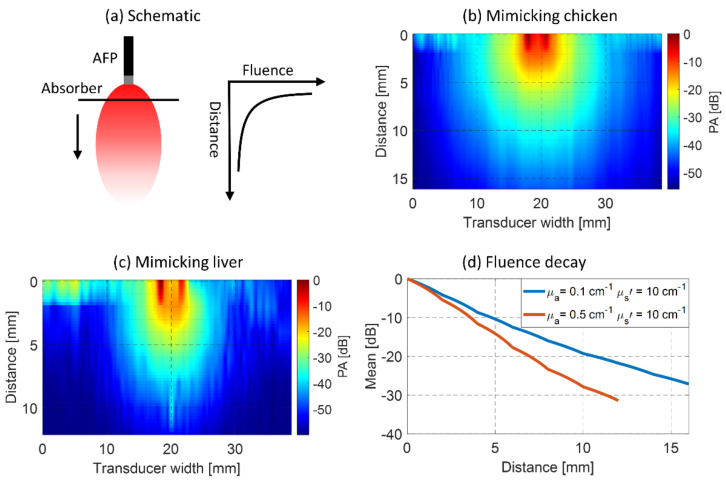
Mean PA magnitude of a black line absorber at several distances from the AFP in a tissue-mimicking phantom. (**a**) Schematic illumination. (**b**) The PA field with optical properties mimicking chicken tissue is shown. (**c**) PA field with optical properties mimicking native liver tissue. (**d**) Mean PA value per depth for both experiments.

For characterizing the AFP, the PA field of view at the distal end was evaluated by measuring the PA signal from a highly absorbing line target (black horse tail hair) at several distances in a liquid phantom, mimicking the optical properties of tissue (see the illustration in Figure 3a). The liquid phantom was a mixture of 20% intralipid (Fresenius Kabi AG, Bad Homburg, Germany) and India ink (Royal Talens, Apeldoorn, the Netherlands) to tune $\mu_{s}\prime$ and $\mu_{a}$. The absorbing line target (named absorber from here) was highly absorbing and 0.19 mm in diameter. The absorber was placed perpendicularly to the AFP and in the focal plane of the US transducer for consistent alignment and maximal PA intensity detection. Illumination of the absorber was performed at 680 nm. US acquisition and light illumination were as explained in the general experimental setup description.

Two experiments were performed, both having a $\mu_{s}^{\prime }$ of 10 cm^−1^, approximately the value for native liver tissue around 680 nm, see [40]. In many experiments, chicken tissue was used to mimic tissue surrounding the liver. Therefore, the first experiment mimics this type of tissue around 680 nm by tuning $\mu_{a}$ to 0.1 cm^−1^ [43]. The second experiment was conducted to mimic liver tissue having a $\mu_{a}$ of 0.5 cm^−1^. During these two experiments, the pulse energy at the distal end of the AFP was 1.0 ± 0.05 mJ.

### 2.3. Validation In Ex Vivo Tissues

#### 2.3.1. Sample Preparation

To mimic the performance of the AFP in a clinical context, ex vivo tissues were used in most of the experiments. Ex vivo chicken breast tissue from a local supermarket was used to mimic tissue surrounding the liver. Ex vivo bovine liver tissue, purchased from a local butcher ~12 h after slaughter, was used to mimic a human liver. Both tissues were stored in a refrigerator prior to the experiment. Contact with water was avoided to minimize alterations in optical properties, and tissues were used within 1–2 days. Human blood purchased from a healthy donor via the blood bank system at the University of Twente was used for imaging of blood inside the liver and for making a tumor phantom. The blood was kept in a cuvette containing heparin as an anticoagulant. Prior to the experiment, it was exposed to air for oxygenation.

#### 2.3.2. Surface Versus Interstitial Illumination

To validate the benefit of illuminating tissue from the inside, PA signals of surface and interstitial illumination were compared. This was performed by determining the PA *SNR* of the trocar and an absorbing target at a shallow depth (15 mm) and at a deeper depth (32 mm). The experiment was performed in chicken tissue. To create an absorbing target, a piece of chicken tissue was dipped into a solution of India ink for 15 min, after which the target was placed first at 15 mm depth and later at 32 mm from the tissue surface. Interstitial illumination and US detection were performed as described earlier. Surface illumination for PA imaging was achieved by coupling light into a 3D printed probe that holds the US transducer with multimode fibers surrounding it [20]. The used wavelength was 720 nm with measured pulse energies at the distal ends of 1.5 ± 0.2 mJ and 9.2 ± 0.3 mJ for interstitial and surface illumination.

#### 2.3.3. Probe and Tine Visualization

Experiments were conducted to both qualitatively and quantitatively verify how the AFP enables visualization of the RFA needle and its tines compared to US imaging alone in chicken and liver tissue. Probe visualization was determined by signals from the AFP and bevel of the RFA device. Tines were defined by their spread, starting from the bevel tip.

The AFP holding the RFA device in its lumen was inserted into the transducer’s imaging plane (in-plane approach). The tines were deployed to the 20 mm mark. The pulse energy used in these experiments was 1.0 ± 0.05 mJ at a wavelength of 680 nm. For quantification, the *SNR* of the tines and the probe were determined. In the first experiment, the imaging capability was tested on ex vivo chicken breast tissue, mimicking the optical properties of tissue surrounding the liver. In the second experiment, fresh ex vivo bovine liver tissue was used.

#### 2.3.4. Blood Vessel Targeting

Mapping blood vessels can help in avoiding their damage by needle insertion. In addition, knowing the location of the tumor relative to blood vessels can potentially help in compensating for the heat sink effect. Therefore, we studied how well blood vessel mimicking tubes appear on PA images after interstitial illumination by the AFP in both chicken breast and ex vivo bovine liver tissue. The tubes were made of nylon and had an inner diameter of 1 mm. Human donor blood was injected into the tubes, which were embedded in tissue a few centimeters from the surface. The AFP was inserted toward the mimicking blood vessel, and the US transducer was placed on the surface to pick up the PA and US signals.

The first experiment was performed in chicken breast tissue, mimicking tissue surrounding the liver. The laser pulse energy was 1.0 ± 0.05 mJ at a wavelength of 760 nm. The AFP was inserted into the transducer’s imaging plane and parallel to the nylon tube. In the second experiment, light with the same wavelength having a pulse energy of 0.7 ± 0.05 mJ was incident on the tube in liver tissue. The AFP was held outside of the transducer’s imaging plane (out-of-plane approach) to realize side illumination on the tube. In the final experiment, the AFP was held in-plane to the transducer and parallelly to the tube, with the bevel of the RFA device slightly inserted. This was performed with a wavelength of 850 nm and 0.6 ± 0.05 mJ of laser pulse energy. For both 760 and 850 nm, human whole blood has a relatively high absorption (2–7 and 4–5 cm^−1^ for deoxygenated and oxygenated blood) with respect to chicken and liver tissue, enabling high PA contrast [35,43,44].

#### 2.3.5. Tumor-Mimicking Target Visibility

The scenario of visualizing a superficial liver tumor from tissue surrounding the liver was mimicked experimentally. A piece of 3 × 1.5 × 1.5 cm chicken breast was dipped into human whole blood for four hours and then embedded in the surface of a piece of ex vivo bovine liver tissue. A piece of chicken breast tissue was placed on top to mimic insertion through the surrounding tissue. The AFP was inserted at an angle parallel to the US transducer for PA imaging. The needle was advanced toward the tumor-mimicking phantom using light at a wavelength of 760 nm and pulse energy of 0.9 ± 0.05 mJ. Since chicken has an absorption coefficient of approximately 0.1 cm^−1^ at 760 nm [43], by dipping it in blood it is expected that the absorption will become approximately that of blood, being 2–7 cm^−1^ for deoxygenated and oxygenated blood at 760 nm [44]. This would mimic positive absorption contrast between chicken and HCC, which has a $\mu_{a}$ of ~1.5 cm^−1^ [25].

#### 2.3.6. Ablated Tissue Targeting

The capability of using interstitial PA imaging with the AFP to visualize an ablation lesion inside liver tissue was explored. A piece of freshly cut ex vivo bovine liver tissue was ablated with a clinical RFA system in which an RF-generator (1500X, Angiodynamics, Latham, NY, USA) generated 150 watts of 460 kHz alternating current. This current was applied through the tines of the RFA applicator device (Starburst XL, Angiodynamics, Latham, NY, USA) through the tissue toward the grounding pad. Chicken tissue was placed on the aluminum bottom of the custom-made tank to homogeneously lead the current from the RFA device over the grounding pad. The tines were deployed up to the 15 mm mark on the trocar. During ablation, a standard procedure for the RFA system was used. The temperature was set to 105 °C and maintained for 5 min.

After ablation, the liver tissue was placed into a container for stable imaging using the AFP. Chicken breast tissue was placed on top of the liver to mimic a layer of soft tissue surrounding the liver. The AFP was inserted through the chicken tissue and advanced toward the ablated region of the liver, during which PA and US images were acquired. The used wavelength was 700 nm with 1.0 ± 0.05 mJ of pulse energy at the AFP’s distal end. This wavelength was chosen since it provided the highest energy output from the OPO.

## 3. Results

### 3.1. Device Testing and Characterization

#### 3.1.1. Monte Carlo Simulation Results

The MCS yields the 3D normalized fluence rate (*NFR*) per pixel in $\frac{W}{W_{0}\cdot {\mathrm{cm}}^{2}}$, $\left[W_{0}\right]$ represents the incident fluence and $\left[\frac{W}{{\mathrm{cm}}^{2}}\right]$ the fluence rate in a pixel. The simulation launches photons from the determined fiber locations into the volume (see Figure 2a). The 3D *NFR*, calculated for 72 fibers illuminating the liver in the native state at 650 nm, can be seen in Figure 2b. The 2D slice shows that fluence from the 72 individual fibers has diffused into a ring-shaped distribution.

To quantify the results of the simulation, the mean of a region of interest (ROI) was taken from a square 1.96 × 1.96 mm (200 × 200 pixels) along the central axis of the AFP for each simulated slice depth. This mean ($NFR_{ROI}$) was converted to $NFR_{rel}\;\left[\%\right]$, being the fraction of the maximum *NFR* ($NFR_{max}$) per slice depth: $NFR_{rel}=\frac{NFR_{ROI}}{NFR_{max}}\cdot 100\;\%$. The homogeneity of the *NFR* distribution within the ROI is then proportional to $NFR_{rel}$. This was performed for the four fiber configurations: 4, 16, 36, or 72 fibers simulated in the annular ring.

Figure 2c–f shows four figures from the simulation results at 650 nm. Figure 2c shows $NFR_{rel}$ for all four fiber configurations and both tissue states with increasing depth. It is seen that for all fiber configurations, the most homogeneous distribution is reached at around 2 mm depth. The percentages were higher when more fibers were included in the simulation since each fiber fires 3 million photons, yet the same optimal depth was found for all configurations. Results from the simulation at 900 and 1050 nm are shown in Supplementary Figure S1, having the maxima 0.5 and 1 mm deeper in native tissue. Interestingly, the optimal depth was found to be at 1.5 mm for ablated tissue at all wavelengths. Figure 2d shows the 2D slice at the found optimal depth at 650 nm, showing a rather homogeneous *NFR* distribution. Figure 2e shows the distribution before the optimal depth and has an annular distribution. Finally, Figure 2f shows a Gaussian distribution since the depth is taken beyond the optimal point. When tissue is ablated, all trends shift to shallower depths. In addition, the maximum values are a few percent higher. This is due to higher scattering, resulting in a slightly more homogeneous spread at the optimal depth.

These results indicate that it takes 1.5–2 mm of depth to reach a homogeneous fluence distribution in front of the AFP, after which a Gaussian profile remains. Due to the annular fluence distribution before the optimal depth, imaging may be less of use in the first 1.5–2 mm.

#### 3.1.2. PA Field Characterization with Absorber

For evaluating the PA field of view with the absorbing line target in the liquid phantom, the mean detected PA magnitude was taken of the absorber at each depth. Linear interpolation was performed between measured distances and in the direction of the transducer. Figure 3 shows these mean values for both optical property settings. In Figure 3a the setup is schematically shown. Results with the chicken tissue-mimicking situation with the absorption of 0.1 cm^−1^ and reduced scattering of 10 cm^−1^ are shown in Figure 3b,c for similar scattering but higher absorption (0.5 cm^−1^), mimicking native liver tissue. The mean PA value per depth from both results is shown in Figure 3d.

In the chicken mimicking phantom (Figure 3b), the PA field is approximately 10–20 mm wide (−35 dB) with a penetration of 10 mm (−20 dB, see Figure 3d). Looking at Figure 3c,d, it is seen that this width is 8–12 mm wide, penetrating 7 mm from the AFP in the liver mimicking phantom. These results show that with increasing absorption, the expected PA field of view gets narrower, and the penetration depth decreases. The lowest field of view is therefore expected to occur when the AFP is used in the liver. The PA magnitude distribution in Figure 3b,c follows a ring-shaped profile in the first and second millimeters from the AFP, after which it converges to a more Gaussian distribution. In Figure 3c, at a 10 mm distance, a PA magnitude is seen that falls outside the observed trends. In the raw data, we see that this is an artifact of unknown origin.

### 3.2. Validation in Ex Vivo Tissues

#### 3.2.1. Surface Versus Interstitial Illumination

**Figure 4:**
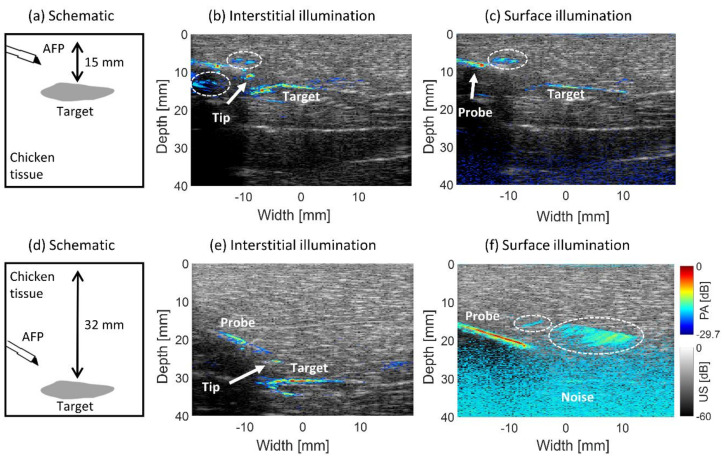
Comparison of interstitial and surface illumination for PA imaging of an absorbing target at 15 and 32 mm depth from the surface. (**a**,**d**) Location of the AFP relative to the target schematically. PA-US images of interstitial illumination (**b**,**e**) and surface illumination (**c**,**f**). The white-dashed circles indicate reconstruction artifacts.

Figure 4 shows the results of imaging an absorbing target in chicken tissue with interstitial illumination using the AFP compared with surface illumination. Figure 4a schematically shows the situation for 15 mm target distance from the surface with the position of the AFP. The overlaid PA-US images for interstitial illumination and surface illumination are shown in Figure 4b,c. The corresponding results for 32 mm target depth Figure 4d are shown in Figure 4d,e.

With interstitial illumination, the absorber’s upper boundary is visible at 15 mm and 32 mm absorber depths, with *SNR*s of 18.7±2.8 dB and 22.2±5.0 dB, respectively. The lower boundary is visible with a lower magnitude. Using surface illumination, only the upper boundary is visible, with a significantly lower *SNR* of 6.4±3.0 dB at 15 mm of depth and with an even lower *SNR* of 2.9±1.0 dB at 32 mm depth. These results show the superiority of interstitial illumination since the fluence propagates the same distance to the target irrespective of its depth from the surface compared to surface illumination.

The tip of the trocar is visible with interstitial illumination at both depths, having an *SNR* of the trocar of 15.1±5.5 dB and 15.0±4.1 dB at 15 mm and 32 mm target depths. With surface illumination, only the upper part of the trocar is seen at 15 mm depth with an *SNR* of 16.0±6.4 dB. At 32 mm depth, the upper and mid-part of the trocar appear in the image, with a homogeneous PA signal from its surface. The *SNR* is found to be 13.7±1.6 dB. Strong reconstruction artifacts occur in all of the images, indicated with the dashed-white lines. Since some parts of the trocar and, more importantly, the tip cannot be visualized with surface illumination, the interstitial method shows superiority since the tip can always be identified, allowing an operator to estimate the probe-absorber distance with greater confidence. In Figure 4f, a large part of the trocar homogeneously generates the PA signal. This is due to the large area of surface illumination, while with the interstitial method, light travels a more forwardly confined beam path.

#### 3.2.2. Probe and Tine Visualization

**Figure 5:**
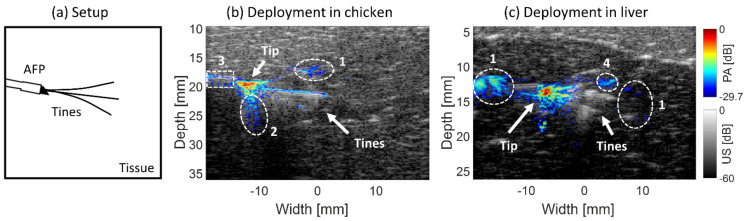
Setup having the AFP with RFA device slightly inserted and tines deployed up to 20 mm in tissue (**a**). Fused PA-US images of the probe and tines in ex vivo chicken (**b**) and bovine liver tissue (**c**). White-dashed lines surround reconstruction artifacts—1, reverberation artifact—2, backscatter signals—3, and a reconstruction artifact appearing to be a tine—4.

PA-US images were created using the AFP with RFA needle slightly inserted and tines deployed to 20 mm inside ex vivo chicken breast and bovine liver tissue, respectively. This is schematically shown in Figure 5a. Looking at chicken tissue in Figure 5b, the probe and its tip are better identifiable in the PA than the US image, with respective *SNR*s of the probe with a bevel of 21.8±8.7 dB and 18.3±0.9 dB. Three tines are visible in both modalities, visible up to approximately 8–10 mm from the bevel. Quantitatively the mean *SNR* of the area encompassing the tines is 6.4±2.3 dB and 5.0±6.8 dB in the PA and US images. Thus, both the probe with its tip and tines are better distinguishable in the PA than the US image. Reconstruction artifacts are encircled by the white-dashed lines numbered 1. The area numbered 2 denotes the reverberation artifact below the bevel tip of the trocar, which is more prevalent in the PA than the US image. Some PA signal originates before the distal end of the AFP due to backscattered light being absorbed by the probe, denoted with encircled area number 3.

Figure 5c shows the visualization in ex vivo bovine liver tissue. Strong PA signal originates from the probe’s tip, with an *SNR* of 21.8±3.2 dB. In the US image, the probe area has a lower *SNR*, being 11.8±0.8 dB. In the PA image, the tines were identifiable up to approximately 4 mm from the probe, with an *SNR* of 13.4±2.2 dB. In the US image, three tines were visible for a greater penetration depth (~10 mm) and higher *SNR*, being 22.2±5.1 dB. These data show that the tip of the probe is easier to identify in the PA than the US image. However, the tines are only visible in the first few millimeters in the PA image, but to a larger extent in the US image. The PA signal observed at 7 mm from the bevel tip overlaps with a tine (seen in US), however looking at the PA image in isolation, it is found to be a reconstruction artifact. This is indicated with the area numbered 4 in the figure.

#### 3.2.3. Blood Vessel Targeting

**Figure 6:**
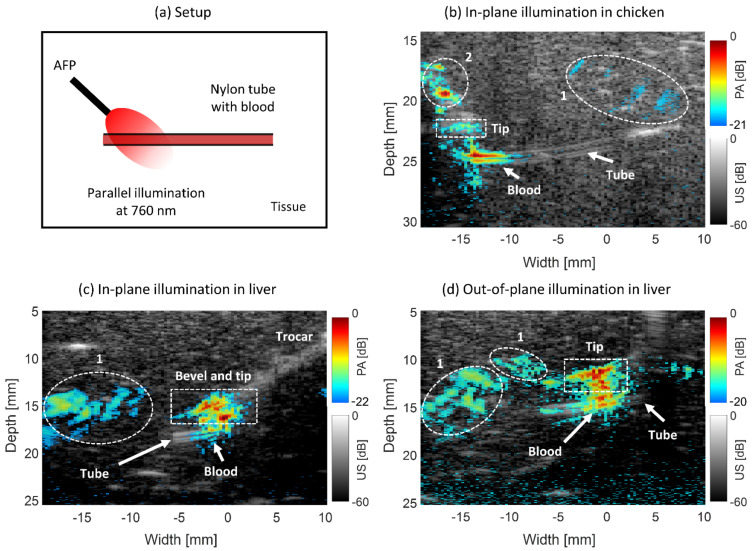
Combined PA-US images of the AFP targeting a nylon tube filled with human blood in chicken breast and ex vivo liver. (**a**) Setup schematically. (**b**) Tube embedded in chicken with illumination in-plane to the US probe. (**c**) In-plane illumination in the liver. (**d**) Out-of-plane illumination in the liver. The white-dashed rectangles indicate the location of the bevel and tip, whereas the circles indicate reconstruction artifacts—1 and PA signals due to light leakage from a fiber in the probe—2. The PA dynamic range is different in b-d for data visibility, as indicated by the color bars.

The PA-US images from guiding the AFP toward a nylon tube filled with human blood are shown in Figure 6. In Figure 6a, the setup can be seen schematically. In Figure 6b, the tube is embedded in chicken tissue and visible in the US image. Looking at the PA signals, approximately 5.5–6 mm long strand of PA signal is coming from the blood with an *SNR* of 12.2±0.8 dB just below the tip. Both the tip of the trocar and its upper part are seen in the PA image contributing to an *SNR* of 11.1±4.5 dB, being quite similar to the *SNR* of the tube. Reconstruction artifacts are present in the images, as indicated with the white-dashed areas numbered 1. The upper PA signal on the trocar in Figure 6b was due to a fiber unintentionally emitting light out of the probe’s wall, numbered 2 in the image. This occurred because the Pebax encapsulation was locally damaged, allowing for some light to escape.

In Figure 6c, the tube was embedded in the liver. The bevel tip of the trocar was slightly inserted, as can be seen by the triangular shape in the upper part of its PA signal. The PA signal coming from the blood (*SNR* of 7.8±0.6 dB) is weaker than that of the tip (*SNR* of 13.2±1.6 dB). The weaker PA signal can be distinguished in two steps, (1) PA signals are located on the tube’s walls in the US image, (2) when moving the AFP slightly up and down, all PA signals move along with it, apart from signals seen in the tube area. The PA signals coming from blood have a width of approximately 4 mm. In Figure 6d, the PA-US image is shown of the AFP illuminating the tube from the side. PA signals coming from the blood are evident in the image with an *SNR* of 10.4±0.4 dB. The tip is also brightly present just on top of the blood vessel with a similar *SNR* of 9.8±1.0 dB. The exact location of the tip is not fully localized since there is a smearing of PA signal, potentially being a combination of reconstruction artifact, the signal from the liver tissue, and the signal from the tip. The width of the PA signal coming from blood is approximately 4 mm, similar to the parallel illumination. This width is not only relying on the penetration depth of light but also on the diameter of the probe since the tube is illuminated rather perpendicularly by the AFP.

Due to the bandwidth of the US transducer (5–11 MHz), only the high-frequency parts of the PA signal from the blood are obtained, which manifests itself by the signal on the tube’s walls.

#### 3.2.4. Tumor-Mimicking Target Visibility

**Figure 7:**
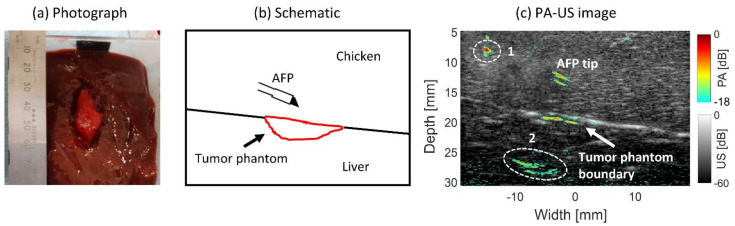
Superficial tumor-mimicking phantom being targeted by the AFP from chicken breast tissue. (**a**) Schematic. (**b**) Photograph of the liver and mimicking tumor. (**c**) Combined PA-US image. The dashed lines: PA signal due to a fiber leaking light—1, and reconstruction artifacts—2.

A human blood-dipped piece of chicken breast was embedded in liver tissue for mimicking a superficial liver tumor targeting through surrounding tissue using the AFP. In Figure 7a, the schematic is shown. In Figure 7b, a photograph of the phantom and liver is shown. The absorption on the rim of the phantom is expected to have similar absorption to blood. The combined PA-US image can be seen in Figure 7c. The AFP device is not visible in the US part of the image, yet the tip is well visible with PA with an *SNR* of 5.6±0.7 dB. The tumor-mimicking boundary is present with a stronger signal having an *SNR* of 7.7±1.9 dB, delineating approximately 7 mm of the boundary in width. The PA signal seems to occur approximately 1 mm below the boundary’s surface, according to the US image. Around 8 mm depth, a PA signal is generated due to a damaged fiber in the AFP shaft and overlapping with the imaging plane of the transducer, as shown by the white-dashed circle, numbered 1. At the bottom, reconstruction artifacts are present, denoted with the white circle and numbered 2. The location of the liver can be best visualized in the US image, being hypoechoic, in contrast to the hyperechoic chicken tissue on top of it. These results indicate that the AFP is able to visualize a superficial mimicking liver tumor with interstitial PA imaging.

#### 3.2.5. Ablated Tissue Targeting

**Figure 8:**
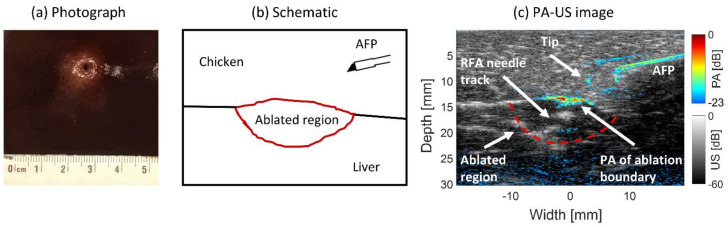
Photoacoustic and ultrasound imaging of the ablation region in ex vivo bovine liver tissue. (**a**) Photograph of the ablated tissue, the whiter area indicates ablated tissue. (**b**) Schematic. (**c**) Combined PA-US image.

Ex vivo bovine liver tissue was ablated with an RFA system and targeted interstitially with the AFP for PA imaging. In Figure 8a, a photograph of the liver tissue with the lesion is shown. Figure 8b schematically shows the position of tissue types during imaging. The photograph shows the ablated region to be whiter in color compared to the red-brown native tissue surrounding it. The middle of the ablation region appears more black due to (1) charring around the electrode and (2) being a hole due to needle perforation. In the whiter area, some brown tint may also be seen, indicating the presence of some charring. Figure 8c shows the PA-US image. The red-dashed curve indicates the location of the ablated region underneath the chicken tissue. The AFP needle shaft is visible, especially in the PA part of the image. There is no PA signal for ~5 mm between the presumable probe tip and the shaft, likely being the metal region of the distal end. The reason for this gap is unclear. As also seen in Figure 4, a reconstruction artifact is present above the needle. The upper ablation boundary shows the PA signal with an *SNR* of 9.76 ± 2.10 dB, being slightly stronger in magnitude than in the US (8.44 ± 0.24 dB). The intensity is highest in the middle, probably due to the presence of charring. The PA signal of the boundary is wider in the depth axis compared to the width in the US image (0.8 vs. 0.5 mm), making the boundary easier to identify in the combined image. The entire upper ablation boundary is visible in the US image, while in the PA image, it is approximately 8.5 mm wide. Total US coverage of the upper boundary is possible because it crosses the oval-shaped ablated lower boundary (red-dashed line). In the US part, the track where the RFA needle was inserted is visible. In addition, the ablated region shows hypoechoic contrast with its native surrounding tissue. The hole in the middle, seen in both modalities, is due to the perforation of the RFA needle.

## 4. Discussion

For successful percutaneous RFA of the liver, accurate localization of the needle and depiction of the extent of the ablation zone is required [8]. Current imaging modalities lack accurate real-time monitoring of the ablation zone during RFA [17,45]. In addition, needle guidance is challenging under US imaging alone [18,19]. Photoacoustic imaging has been proposed as a potential technique to overcome these challenges [20]. To overcome the low penetration depth of light in tissue, we investigated interstitial illumination for PA guidance of RFA. For this, we designed the AFP, a probe that allows sliding an RFA device through its lumen, with optical fibers at the distal end for interstitial illumination. A conventional US transducer detects the generated PA signals from the RFA device and tissue. In this section, the results of the probe’s characterization, detection of structures, and needle guidance are discussed.

### 4.1. Device Testing and Characterization

#### 4.1.1. Monte Carlo Simulations

To investigate the relation between the number of fibers at the distal end in the probe and the fluence distribution, MCS was performed with 4, 16, 36, and 72 fibers arranged in the probe. It was observed that the fluence distribution converges from hotspots into an annular distribution. However, the annular distribution did not occur with four fibers. The higher the number of fibers used, the less depth is required to reach this annular shape, as also observed in work by Eddins and Bell (2017) [38]. They also found that light from the tool tip converges to a Gaussian illumination spot at further depth, which is important for proper device guidance and imaging of the region of interest. We observed similar behavior in the simulation; for all numbers of fibers, the most homogeneous fluence distribution occurs approximately at 2 mm depth from the probe for native tissue and at 1.5 mm for ablated tissue (Figure 2). This decrease in depth is likely due to the higher scattering in ablated tissue, lowering the mean free path [34,37]. The difference of this depth at different wavelengths was lower in ablated tissue. This phenomenon can also be attributed to the higher scattering. These results indicate that in the first few millimeters in front of the AFP, PA signals are primarily expected to come from a circular region within the tissue, whereas at further depths, signals will also originate from the entire surface filling the lumen in front of the AFP. In addition, since each fiber launches 3 million photons, the most homogeneous fluence distribution is found with 72 fibers in the probe. This mimics non-optimal coupling at the proximal side since the total fluence is increased when fibers are added in the simulation.

#### 4.1.2. PA Field Characterization with Absorber

We measured the PA field of view of the AFP in a liquid phantom. An annular-shaped PA distribution in the first 2 mm from the probe was observed, similar to the fluence distribution found in the MCS. There was an additional PA signal in the middle of the annular distribution, of which the origin is unclear. The results also indicate that PA signals in native liver tissue can be expected up to 7 mm from the AFP (−20 dB). This means that assessing an entire ablation volume in the liver will be challenging since RFA is used to ablate tumors up to 3 cm in diameter [3,45]. Although the PA penetration depth in ablated tissue may be different, it will not cover an ablation diameter. This might be overcome by advancing the AFP through the ablation volume to assess its diameter. Another approach could be placing a cylindrical diffuser-based illumination fiber through the ablation volume, which is capable of illuminating tissue homogeneously along its distal end [46].

### 4.2. Validation in Ex Vivo Tissues

#### 4.2.1. Surface Versus Interstitial Illumination

With interstitial PA imaging, the low optical penetration depth in tissue is overcome by bringing light to the location of interest [34]. We compared the efficacy of PA imaging with interstitial and surface illumination. The signal strength of the absorbing boundary at different depths was similar with interstitial illumination but different with surface illumination. With interstitial illumination, the tip was always identifiable, which was not the case with surface illumination for the deeper-lying target, as was seen in our previous work with interstitial PA imaging [28]. This is supporting evidence that interstitial illumination is superior to surface illumination in finding the location of the needle tip and the border of a target at locations farther than the penetration depth of light from the tissue surface, showing the added value of using interstitial PA image-guided interventions. In addition, laser power can be maintained relatively constant with increasing target depth using interstitial illumination, while with surface illumination, an increase is often required, which is limited by the maximal permissible exposure in tissue [47].

#### 4.2.2. Probe and Tine Visualization

It has been shown that PA imaging increases needle tip visualization compared to US imaging [48]. Our results showed as well that tip visibility was superior in the PA image, making the tip easier to identify in the fused PA-US image. Tine visualization was not superior with PA imaging, being comparable with the US in chicken tissue, but of lower magnitude in liver tissue with PA imaging. Due to differing optical properties, the tines could be distinguished at a greater distance from the AFP in the PA image in chicken tissue (~9 mm) than in liver tissue (~4 mm) [40,43].

#### 4.2.3. Blood Vessel Targeting

In the work of Kempski et al. (2019), it was shown that in vivo hepatic veins could be visualized using PA imaging [24]. Real-time visualization of blood vessels would have potential added value for the safe insertion of RFA needles and improved treatment outcomes. In our previous work, we performed imaging of human blood in chicken tissue with surface illumination [20]. In this work, we extended our findings with interstitial illumination in chicken and ex vivo liver (Figure 6). We found that the tip of the AFP probe and the bevel of the trocar were always visible with PA imaging, signals from blood were also present, yet they were harder to localize since the signal of the tip can overshadow the presence of blood, especially in the liver. In addition, the PA detectable area of blood was only a few millimeters wide due to the low penetration depth of light. However, the presence of blood could be confirmed by moving the probe up and down, leaving the PA signal of blood at the same location.

The vascular network can be assessed in the liver using US **[49,50]**, therefore using our method to combine interstitial PA imaging with US imaging, more accurate tip localization relative to blood vessels can be obtained. For distinguishing blood from other absorbers, multi-wavelength PA imaging may provide added value. Due to the bandwidth of the US transducer (5–11 MHz), only the high-frequency parts of the PA signal from the blood were obtained, showing only the sharp discontinuities of the signal, which coincide with the (inner) walls of the tube [51].

#### 4.2.4. Tumor-Mimicking Target Visibility

The most prevalent tumors in the liver are hepatocellular carcinoma (HCC) and colon liver metastases (CLM) [1,2]. Screening for HCC is often performed with US. Diagnosis may also be conducted with this technique [52]. It is interesting to explore if PA imaging could aid in detecting tumor tissue during insertion with the RFA device using the AFP. We found that the boundary of a superficial HCC phantom (a blood-dipped piece of chicken tissue) was detectable through surrounding mimicking tissue at 760 nm with interstitial PA imaging (Figure 7). PA signals occurred ~1 mm below the phantom boundary seen in the US image. It is not known to what extent blood has actually penetrated beyond the tissue rim. Therefore, it is uncertain if the location of the PA signal is due to the blood penetration profile or that this phenomenon has a different origin.

Our phantom, embedded in the surface of ex vivo bovine liver tissue, mimics the positive optical absorption contrast between surrounding tissue and a superficial HCC [43,44]. The phantom is not mimicking the optical absorption contrast between HCC surrounded by liver tissue, since absorption at 760 nm is lower in HCC compared to healthy liver tissue (~1.5 cm^−1^ vs. 2.0 cm^−1^, respectively) [25].

#### 4.2.5. Ablated Tissue Targeting

In ex vivo bovine liver tissue, the *SNR* of the upper ablation boundary was 1.3 dB higher in the PA image than in the US image. The lower boundary was only visible in the US but with a less clear transition than the upper boundary (Figure 8). The PA signal demarks the boundary more clearly than the US, and therefore, the PA-US image allows more accurate detection of the ablation boundary while allowing structural information with the US. The main limitation is the low penetration depth of light, making it necessary to target the AFP close to the boundary for it to be detected and have a limited response in the horizontal direction. Due to this, ablation diameter assessment can only be performed by sliding the AFP through the volume. Another approach is using the earlier mentioned diffuser fiber, which can illuminate the diameter of a small ablation site [46]. In the middle region, a black color (char) was observed, which has higher absorption than coagulated tissue and therefore positively affects the strength of the PA signal [53]. For accurate assessment of ablation, targeting the ablation boundary should be accompanied by distinguishing native from ablated tissue. Therefore, in future work, we will explore multi-wavelength PA imaging for visualizing the differences in optical properties between native and ablated tissue [40].

#### 4.2.6. Laser Fluence

The maximum used pulse energy with the AFP was 1.7 mJ at 720 nm, resulting in a fluence at the distal end of 122 mJ cm^−2^, exceeding the ANSI limit of 21 mJ cm^−2^ for skin radiation at the given wavelength [47]. However, laser-related damage to the tissue was not observed with 3–5 min of exposure. This observation agrees with the recent finding of Huang et al. (2021), who showed in swine livers that laser damage to tissue did not occur during a 10-min exposure to 150 mJ cm^−2^ of pulsed laser light at 750 nm [54]. While these aspects need to be studied in more detail, if there is a requirement for the use of the AFP for long periods, then a recommendation would be to intermittently switch off the PA imaging mode.

#### 4.2.7. Reconstruction Artifacts

Most PA images in this work have multiple artifacts, especially reconstruction artifacts. They manifest as smearing of the PA signal or a diverging copy of the needle. This artifact was similarly observed in other works [19,20,27]. In our previous work [20], the artifact was identified as a filtered back-projection artifact appearing with high-intensity PA signals such as from the tip in this work.

While the artifacts in static PA images presented in this paper may look overwhelming, when working with the system in real-time, it becomes evident which PA signals are from tissue structures and which are from artifacts. The artifact typically moves with needle insertion and often appears as a smear distant from the tip, while PA signals from tissue chromophores remain static with insertion. In addition, during probe insertion, the PA images are compared with the co-registered US images for better context and orientation.

## 5. Conclusions

A probe with optical fibers in an annular distribution for interstitial illumination photoacoustic (PA) imaging was designed and tested for its potential in image-guided radiofrequency ablation (RFA) of the liver. Light exiting the probe has an annular distribution and requires approximately 2 and 1.5 mm of depth in native and ablated liver tissue to reach homogeneous illumination. Interstitial illumination was superior to surface illumination in PA imaging, having comparable PA signal strength from the tip of the needle and a target irrespective of depth from the tissue surface. Using the AFP, the location of the needle tip was visible at all times with higher signal strength in the PA image than in the ultrasound (US) image. PA signal from human blood in nylon tubes was visible in bovine liver and chicken tissue; however, exact localization was challenging due to the high PA signal from the nearby needle tip. RFA needle insertion, accompanied by interstitial PA imaging combined with US imaging, could allow better tip localization and avoidance of blood vessels. We also showed that the boundary of a tumor-mimicking phantom and an ablation region in bovine liver were detectable with PA imaging. However, multi-wavelength PA imaging should be explored to discriminate healthy and native liver tissue from tumor and ablated tissue. In addition, since light penetration in tissue is low, the PA imaging depth with the probe was only 9 mm in chicken and 4 mm in liver tissue, respectively, finetuning the illumination strategy should mitigate this limitation. Our AFP shows potential to aid in targeting and monitoring RFA of the liver using interstitial illumination PA imaging.

## Figures and Tables

**Table 1:** Optical parameters used in the Monte Carlo simulations. The anisotropy factor g was estimated using reference [35]. The absorption $\mu_{a}$ and reduced scattering coefficient $\mu_{s}\prime$ were taken from reference [40]. The refractive index n was estimated from reference [41] and was kept similar for both tissue types. The reduced scattering coefficient was determined by $\mu_{s}=\mu_{s}^{\prime }/\left(1-g\right)$.

Optical Parameter	Native Liver Tissue			Ablated Liver Tissue at 70 °C		
Wavelength $\left[\mathrm{nm}\right]$	650	900	1050	650	900	1050
$\mu_{a}\;\left[{\mathrm{cm}}^{-1}\right]$	1.23	0.73	0.60	0.70	0.23	0.24
$\mu_{s}^{\prime }\;\left[{\mathrm{cm}}^{-1}\right]$	8.26	5.04	3.85	31.48	24.90	18.341
$\mu_{s}\;\left[{\mathrm{cm}}^{-1}\right]$	118.01	72.03	55.02	314.79	248.98	183.41
g	0.93	0.93	0.93	0.90	0.90	0.90
n	1.33	1.33	1.33	1.33	1.33	1.33

## Data Availability

The data presented in this study are available on request from the corresponding author.

## Supplementary Materials

The following is available online at https://www.mdpi.com/article/10.3390/s21134458/s1, Figure S1: *NFR_rel_* resulting from the Monte Carlo simulations for 650, 900, and 1050 nm.
