# Supplementary material for: Annular Fiber Probe for Interstitial Illumination in Photoacoustic Guidance of Radiofrequency Ablation
Source: Sensors (Basel). 2021 Jun 29;21(13):4458. doi: 10.3390/s21134458 (PMC8271966; doi:10.3390/s21134458)
Supplement: Supplementary file 1 [file sensors-21-04458-s001.zip › sensors-1250370-supplementary.pdf]

Supplementary Material

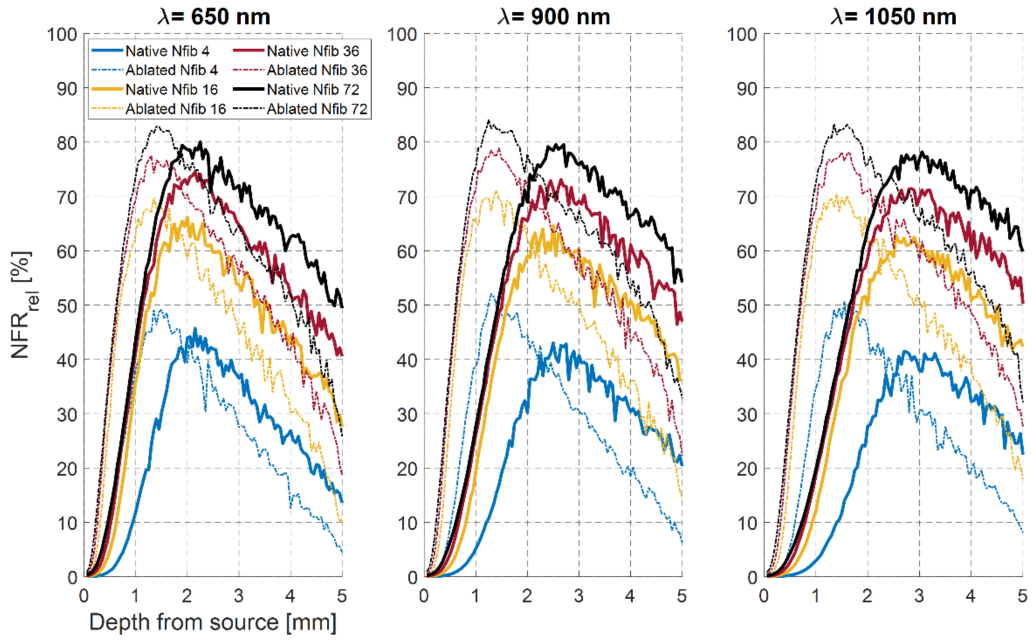

Figure S1. NFR_{rel} from the Monte Carlo simulations of native and ablated liver tissue at 650 nm, 900 nm, and 1050 nm for 4, 16, 36 and 72 fibers in the annular ring. It is seen in the three figures that 1) the optimum does not significantly depend on the number of fibers in the ring. 2) At 650 nm the optimum for native tissue is approximately at 2 mm, while it is 2.5 mm and 3 mm for 900 nm and 1050 nm respectively. 3) In ablated tissue, the optimum is always found around 1.5 mm.
